# ITRAQ-based quantitative proteomics reveals the proteome profiles of MDBK cells infected with bovine viral diarrhea virus

**DOI:** 10.1186/s12985-021-01592-2

**Published:** 2021-06-06

**Authors:** Yaxin Li, Tao Guo, Xiaokui Wang, Wei Ni, Ruirui Hu, Yuying Cui, Taotao Mi, Shengwei Hu

**Affiliations:** College of Life Sciences, Shihezi University, Shihezi, 832003 Xinjiang China

**Keywords:** BVDV, Virus infection, Proteomics, iTRAQ, MDBK cells

## Abstract

**Background:**

Bovine viral diarrhea (BVD) which is caused by Bovine viral diarrhea virus (BVDV), is an acute, contagious disease. In spite of the use of vaccines and elimination projects, BVDV still causes severe economic losses to the cattle industry for the past few years. The current study presents a preliminary analysis of the pathogenic mechanisms from the perspective of protein expression levels in infected host cells at different points in time to elucidate the infection process associated with BVDV.

**Methods:**

We used the isobaric tags for relative and absolute quantitation (iTRAQ) technology coupled with liquid chromatography-tandem mass spectrometric (LC–MS/MS) approach for a quantitative proteomics comparison of BVDV NADL-infected MDBK cells and non-infected cells. The functions of the proteins were deduced by functional annotation and their involvement in metabolic processes explored by KEGG pathway analysis to identify their interactions.

**Results:**

There were 357 (47.6% downregulated, 52.4% upregulated infected vs. control), 101 (52.5% downregulated, 47.5% upregulated infected vs. control), and 66 (21.2% downregulated, 78.8% upregulated infected vs. control) proteins were differentially expressed (fold change > 1.5 or < 0.67) in the BVDV NADL-infected MDBK cells at 12, 24, and 48 h after infection. GO analysis showed that the differentially expressed proteins (DEPs) are mainly involved in metabolic processes, biological regulation and localization. KEGG enrichment analysis showed that some signaling pathways that involved in the regulation of BVDV NADL-infection and host resistance are significantly (P < 0.05) enriched at different stages of the BVDV NADL-infection, such as Endocytosis signaling pathway, FoxO signaling pathway, Homologous recombination signaling pathway and Lysosome pathway.

**Conclusions:**

These results revealed that the DEPs in BVDV NADL-infected MDBK cells have a wide range of regulatory effects; in addition, they provide a lot of resources for the study of host cell proteomics after BVDV infection.

**Supplementary Information:**

The online version contains supplementary material available at 10.1186/s12985-021-01592-2.

## Background

According to the cytopathic condition in the cell, Bovine viral diarrhea virus (BVDV) was divided into cytopathic type (CP) and non-cytopathic type (NCP) [1]. The virus can also spread from species to species, infecting a variety of animals such as pigs, sheep, deer and camels [2]. Although known as diarrhea disease, the harm to cattle is not limited to diarrhea, but also serious effects on the reproductive system, respiratory system, digestive system, and milk production occur [3]. The disease may be spread by direct or indirect contact. Pregnant cows infected with BVDV can infect fetuses through the placenta, causing miscarriage and congenital loss of calves, or they may give birth to seemingly normal but persistently infected animals. BVDV is widespread worldwide, and in spite of the use of vaccines and eradication programs, it still causes serious economic losses to the breeding industry. In recent years, the epidemiological surveys have found that the prevalence of BVDV in large cattle farms in China continued to rise, and the antibody positive rate in some cattle farms was even as high as 90%.

With the development of proteomics technology, it has outstanding advantages in studying virus-host cell interactions. However, there is very limited research on the proteomic information of MDBK cell line responses to BVDV NADL infection. Proteomics was originally proposed by Australian scholar Wilkins at the bidirectional electrophoresis conference in 1994. From the study of intracellular protein composition, overall horizontal activity patterns and protein interactions, high-throughput analysis of multiple species samples by proteomics technology allows for the constitutive expression, quality assessment levels and modification status of proteins in the samples. In this way, the function of proteins and the potential relationship between proteins can be revealed and new proteins can be discovered. Proteomics method can be used to analyze proteins on a large scale effectively, which is more helpful for the in-depth exploration of the pathogenesis of various diseases. Quantitative proteomics approaches can be used to screen and find the deferentially expressed proteins between samples caused by any factor, mainly for the relative quantitative analysis of protein expression differences in cells or tissues, such as difference gel electrophoresis (DIGE) [4], sequential windowed acquisition of all theoretical fragment ions (SWATH). Combined with bio-informatics to reveal the physiological and pathological functions of cells, it can also qualitatively and quantitatively analyze certain key proteins.

Isobaric tags for relative and absolute quantification (iTRAQ) coupled with tandem mass spectrometry (LC–MS/MS) has become a more powerful method for quantifying proteomics because it is more sensitive and precise than conventional proteomics methods [5], especially for detecting low-abundance protein quantification in samples. In recent years, the quantitative proteomics techniques of iTRAQ have been applied to the study of virus-host interactions [6–8]. However, few studies have been conducted on proteomic changes in MDBK cells after BVDV NADL infection using high-throughput methods.

In our study, isobaric tags for iTRAQ coupled with LC–MS/MS quantitative proteomics technology was used to study and analyze the DEPs of MDBK cells which were infected by BVDV NADL, to show the changes in host response to virus invasion after BVDV NADL infection and reveal the pathogenesis of virus infection. The results not only enhance our understanding of the BVDV-host interaction at the protein-level, but also point the way to potential antiviral targets.

## Methods

### Cell culture

The MDBK cell line (Free of BVDV and anti-BVDV antibodies) used in this study was purchased from the Chinese Academy of Sciences Type Culture Collection (Shanghai, China) and stored in Animal Genetic Engineering Laboratory, Shihezi University School of Life Science (Shihezi, China). The stored MDBK cells were removed from the liquid nitrogen and melted rapidly at 37 °C. The cells were cultured in Dulbecco's modified Eagle's medium (DMEM; Gibco, China) with 10% fetal bovine serum (FBS; Hyclone). All of the materials for this study are kept out of BVDV and anti-BVDV antibodies. BVDV standard strain NADL was obtained from the State Food and Drug Administration (Beijing, China).

### Virus inoculation

When the bottom surface was covered by the above cultured MBDK cells (aggregate ~ 75–85%), we changed the DMEM with 10% FBS to the medium without serum and the cells were infected with BVDV NADL (100 TCID50/ 0.1 ml). Therefore, we required 50% of tissue culture infective dose (TCID50) of BVDV NADL to be prepared before infection. When the infection was processed for two hours, we provided the culture with fresh medium containing serum (5% FBS). These cells were gathered at 12 h, 24 h and 48 h after they were infected. We provide the same amount of PBS to the control group. We verified the BVDV infection through observing the cytopathic effect (CPE).

### Sample preparation, protein digestion and iTRAQ labeling of peptide

We collected the BVDV infected at 12, 24 and 48 hpi as well as uninfected MDBKs with a cell scraper, which is followed by centrifuging at 1500 × g for 10 min and washed with PBS. The collected cells were lysed with lysis buffer which contained 100 mm NH_4_HCO_3_ (pH 8), 6 M Urea and 0.2% SDS, and they were followed by 5 min of ultrasonication on ice. Then, the lysate was centrifuged at 12000xg for 15 min at 4℃ and we took the supernatant to a clean tube. Extracts from samples were reduced with 10 m MDTT for 1 h, and subsequently alkylated with sufficient iodoacetamide for 1 h at room temperature in the dark. Then samples were completely mixed with precooled acetone by vortexing and they were incubated at – 20 ℃ for at least 2 h. Samples were then centrifuged and the precipitation was collected. After we washed it twice with cold acetone, the pellet was dissolved by dissolution buffer. They contained 0.1 M triethylammonium bicarbonate (TEAB, pH 8.5) and 6 M urea, and then the concentration was determined [9]. The protein solution (120 μg) was diluted with 50 mM TEAB in proportion. After digestion with trypsin Gold, peptides were desalted with a Strata X C18 column that was used to make the peptides desalted. After that, they were desiccated and then dissolved in 20 μl 1 M TEAB. Peptide labeling was performed with iTRAQ Reagent 8-plex Kit following the manufacturer's instruction.

### Peptide fractionation and LC–MS/MS

All labeling samples were mixed with equal volume, desalted and lyophilized. Mobile phase A and B were applied to be a gradient elution. The sample was fractionated with a C18 column (Waters BEH C18 4.6 × 250 mm, 5 μm) on a Rigol L3000 HPLC system, and the column oven was set as 50 °C. The eluates were monitored at UV 214 nm. They were collected for a tube per minute and combined into 10 fractions. Most of the fractions were dried under vacuum, and then, reconstituted in 0.1% (v/v) formic acid (FA) in water.

The sample was injected into a home-made C18 Nano-Trap column (2 cm × 75 μm, 3 μm). Then, we separated the peptides in a home-made analytical column (15 cm × 150 μm, 1.9 μm) by the way of using a linear gradient elution. The separated peptides were analyzed through Q Exactive HF-X mass spectrometer (Thermo Fisher). It also worked with ion source of Nanospray Flex™(ESI), spray voltage of 2.5 kv and ion transport capillary at the temperature of 320 °C.

### Data analysis

The resulting spectra from each fraction were searched separately against the Bos taurus database (ftp://ftp.ensembl.org/pub/release-99/fasta/bos_taurus/dna/Bos_taurus.ARS-UCD1.2.dna_rm.toplevel.fa.gz). The identified protein contains at least 1 unique peptide with FDR less than 1.0%. Reporter Quantification (iTRAQ 8-plex) was used for iTRAQ quantification. The protein quantitation results were statistically and correctly analyzed by Mann–Whitney Test. Because the quantitative results of some proteins were observably different between the experimental group and the control group, (p < 0.05 and FC > 1.5 or < 0.67 ([fold change, FC]), these proteins were defined as differentially expressed proteins (DEPs).

### Bioinformatic analysis

All differential proteins were mapped to each term in the Gene Ontology database (http://www.geneontology.org/) using the interproscan-5 program [10], the number of proteins in each term was calculated, and then hypergeometric tests were applied to find GO entries significantly enriched in differential proteins compared with all protein backgrounds [11]. Pathway analyses were extracted using the search pathway tool in the KEGG Mapper platform (http://www.genome.jp/kegg/mapper.html) [7]. The StringDB protein interaction database (http://string-db.org/) was used to carry out interaction analysis of identification proteins. Then blast comparison was carried out between the sequences of differential proteins, and the sequences were extracted to obtain corresponding interaction information, so as to construct a network diagram [12].

## Results

### Confirmation of BVDV NADL Infection in MDBKs

**Fig. 1:**
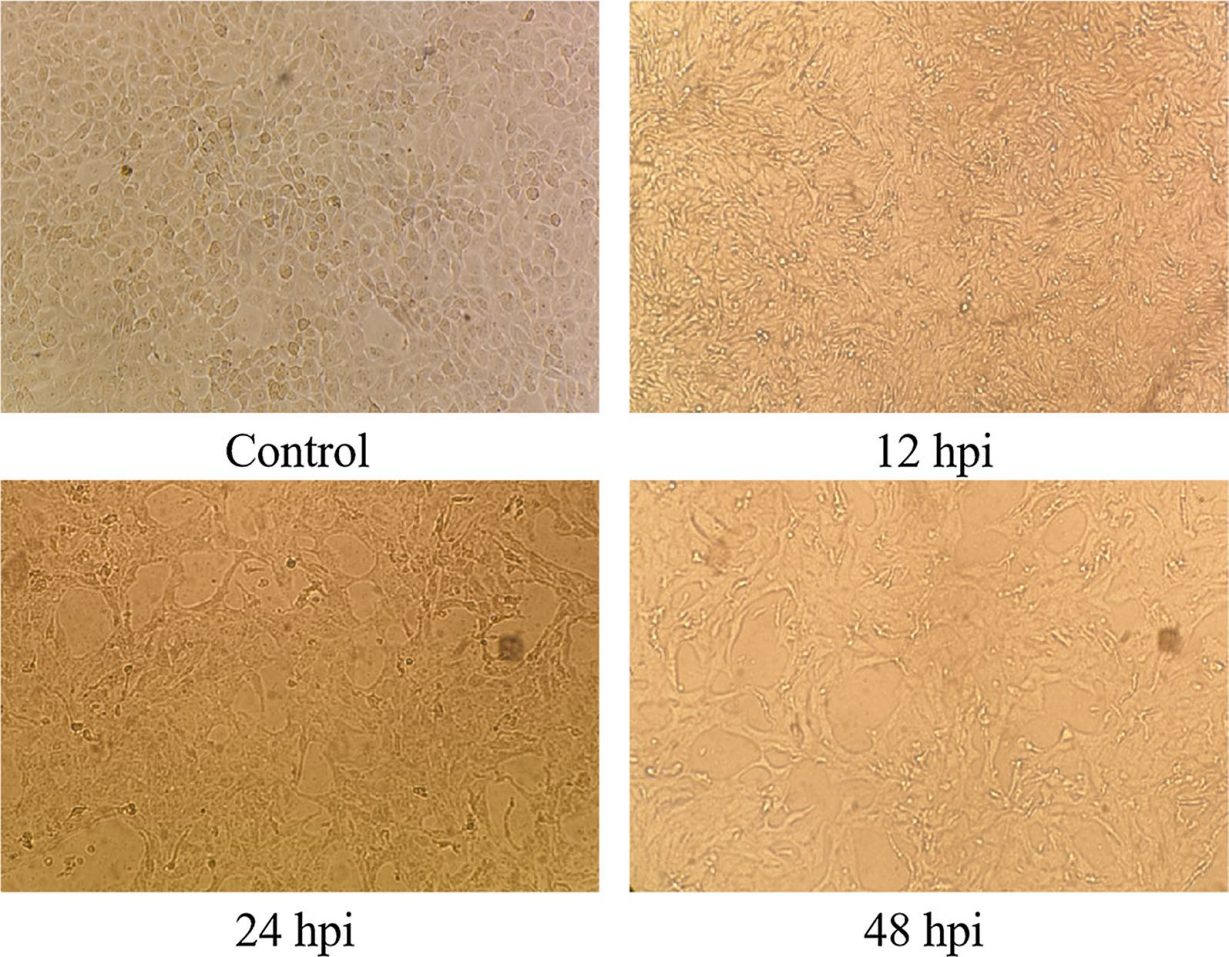
BVDV NADL infection in MDBKs. The cytopathic efects (CPE) of MDBKs at 12, 24, and 48 h after infection, and mock-infected cells as control

Successful BVDV NADL infection was verifed by observation of the cytopathic effect (CPE). The results were presented in Fig. 1. After MDBK cells were infected, aggregation of cells was observed under an inverted microscope at 12 hpi, and more obvious cytopathic changes were about to occur. At 24 hpi after infection, severe cytopathic changes were observed, including aggregation and rounding of cells, and the shedding of some cells resulted in the pulling network. The longer the virus infected cells, the more severe the cytopathic changes were, and eventually all cells died and fell off (Fig. 1).

### Identification of differentially expressed proteins by iTRAQ LC–MS/MS analysis

**Fig. 2:**
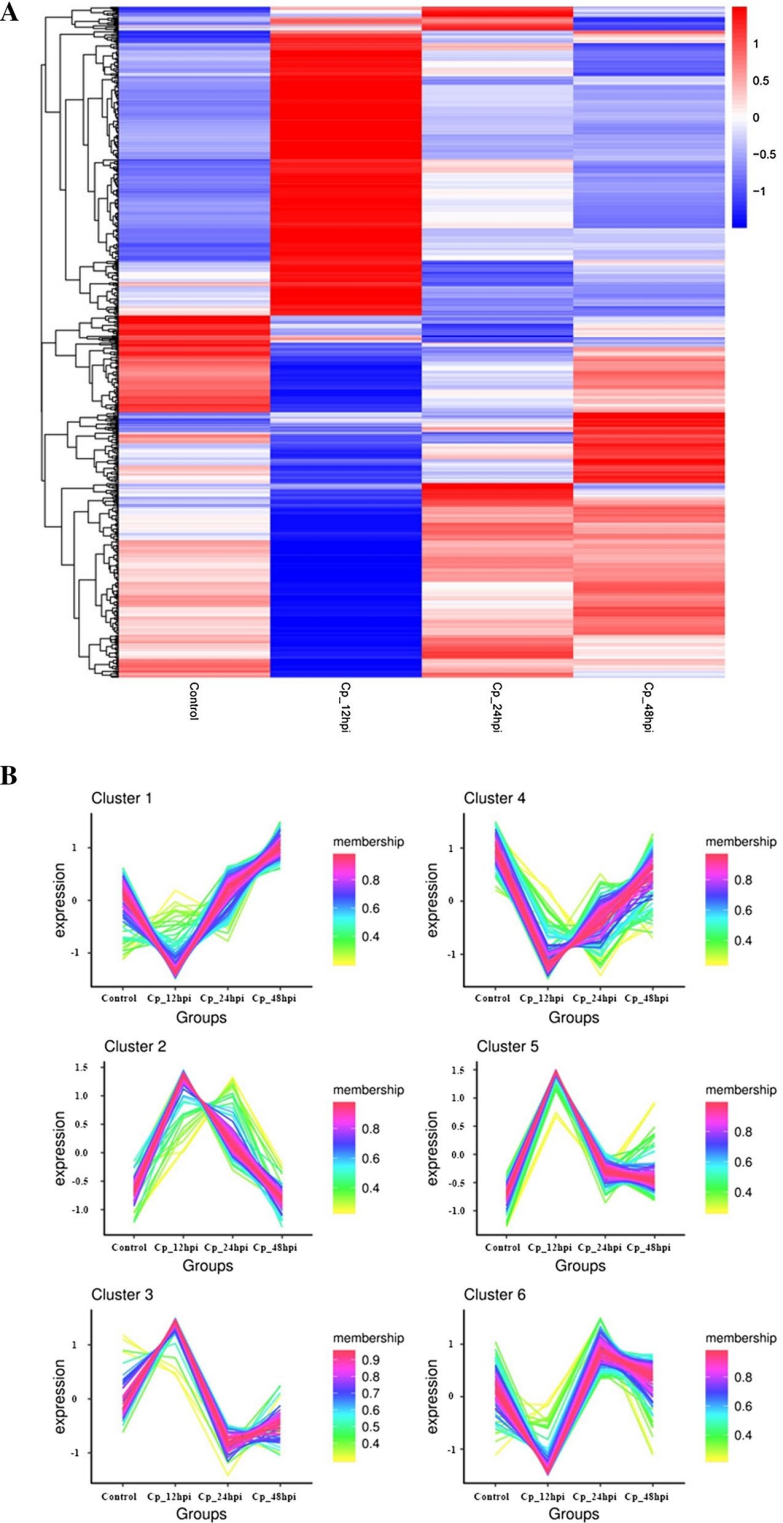
Identification of dysregulated proteins in BVDV NADL-infected MDBK cells by iTRAQ LC–MS/MS Analysis. **a** Hierarchical Clustering Analysis of differentially expressed proteins. Clustering with log 10 (TPM + 1) values, red for high expression proteins, blue for low expression proteins. **b** C-means cluster diagram of differentially expressed proteins. The x-coordinate is the grouping, and the y-coordinate is the z-value correction of the expression quantity. The larger the expression quantity is, the higher the expression quantity is; otherwise, the lower the expression quantity is. Each polyline represents 1 protein, and the color represents the size of the value. The larger the value, the closer the protein is to the average level in this classification

Considering the dynamic changes in host response to viral infection, we collected the whole cell lysates of MDBK cells at 12 hpi, 24 hpi, and 48 hpi In this study, the differentially expressed proteins were detected and their relative rates of change were analyzed. Totally, 3191 proteins were found in the BVDV NADL-infected and mock-infected groups (Additional file 1: Table S1). Through controlling fold change more than 1.5 or less than 0.67 and p value < 0.05, we obtained the differentially expressed proteins in the BVDV NADL-infected MDBK cells (Additional file 2: Table S2). Among the proteins that show significantly different levels of expression after infection, 357, 101, and 66 proteins were differentially expressed relative to uninfected MDBK cells at 12, 24, and 48 hpi. Of the 357 DEPs at 12 hpi, 187 proteins were upregulated and 170 proteins were downregulated. While 354 and 108 proteins were differentially expressed relative to the MDBK cells that were infected for 48 h at 12 hpi and 24 hpi. Moreover, we also showed the hierarchical clustering analysis and the C-means clustering diagram in Fig. 2a, b.

### GO analysis of differentially expressed proteins

**Fig. 3:**
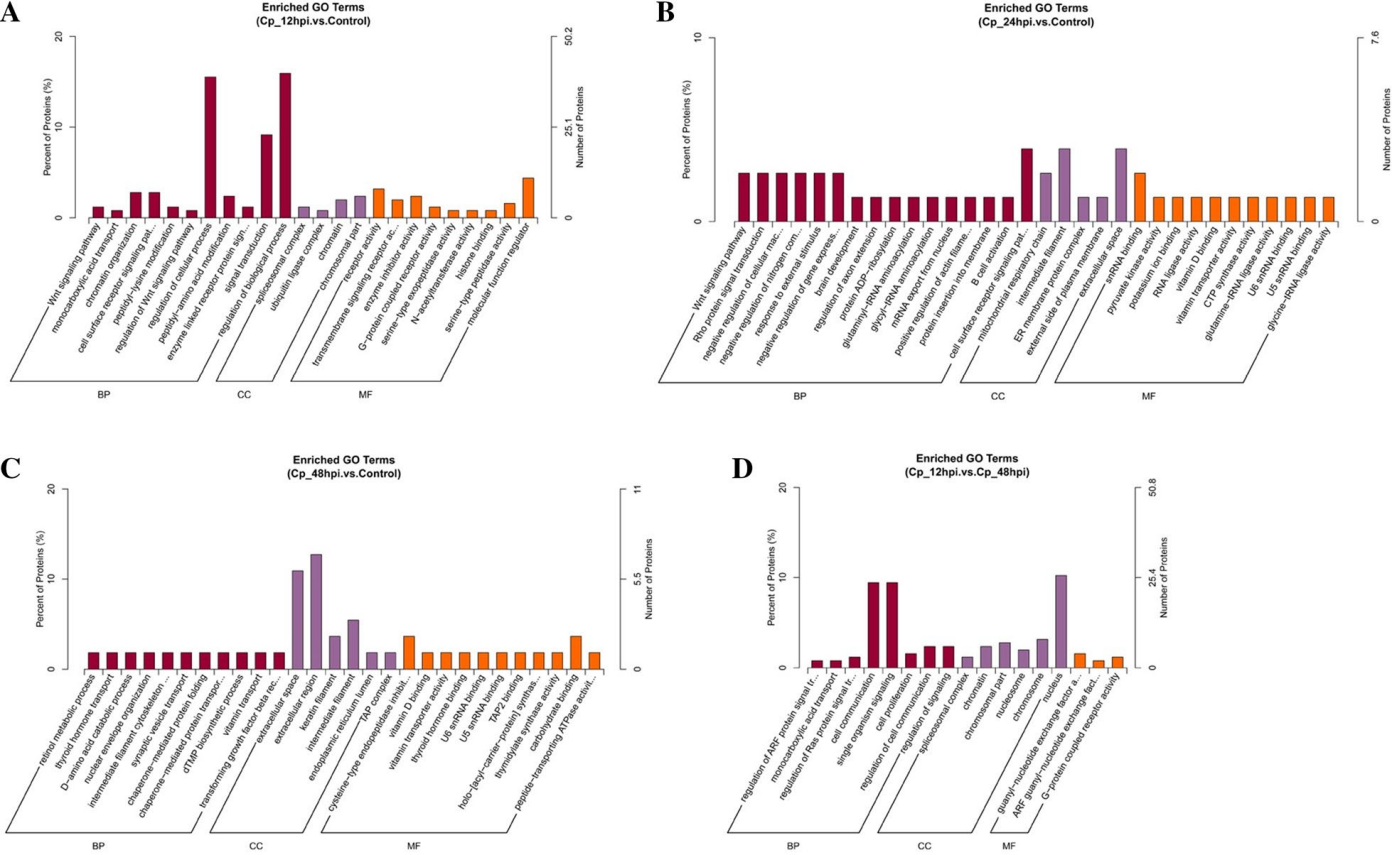
GO analysis of differentially expressed proteins. **a**–**d** GO analysis was performed on the differentially expressed proteins in Cp_12hpi vs. control (**a**), Cp_24hpi vs. control (**b**), Cp_48hpi vs. control (**c**) and Cp_12hpi vs. Cp_48hpi (**d**). The abscissa is the GO macromolecules and GO term at the next level, and the ordinate is the number and proportion of genes annotated to the term

To understand the possible biological functions of the dysregulated proteins in BVDV NADL infection, both up-regulated and down-regulated proteins at 12, 24 and 48 hpi were done with the gene ontology (GO) analysis (Additional file 2: Table S2). The results showed that 320, 165 and 94 terms were prominently enriched in biological processes (BP), cellular components (CC), and molecular functions (MF) at 12, 24 and 48 hpi, respectively. Especially at 12 hpi, the processes of MBDK cells are found the most active. For the dysregulated proteins at 12 hpi, most of the differentially expressed proteins are concentrated in the regulation of biological process (BP) and cellular process (BP), signal transduction (BP), chromosomal part (CC), and molecular function regulator (MF) (Fig. 3a). Whereas cell surface receptor signaling pathway (BP), intermediate filament (CC), extracellular space (CC), and snRNA binding (MF) were mostly enriched at 24 hpi (Fig. 3b). And then for the discrepant proteins at 48hpi, the most enriched GO terms were extracellular region (CC), intermediate filament (CC) and cysteine-type endopeptidase inhibitor activity (MF) (Fig. 3c). On the other hand, differentially expressed proteins in the early stage of infection (Cp_12hpi) compared with those in the late stage of infection (Cp_48hpi) are concentrated in the cell communication(BP), single organism signaling (BP) and nucleus (CC) (Fig. 3d).

### KEGG pathway analysis of differentially expressed proteins

**Fig. 4:**
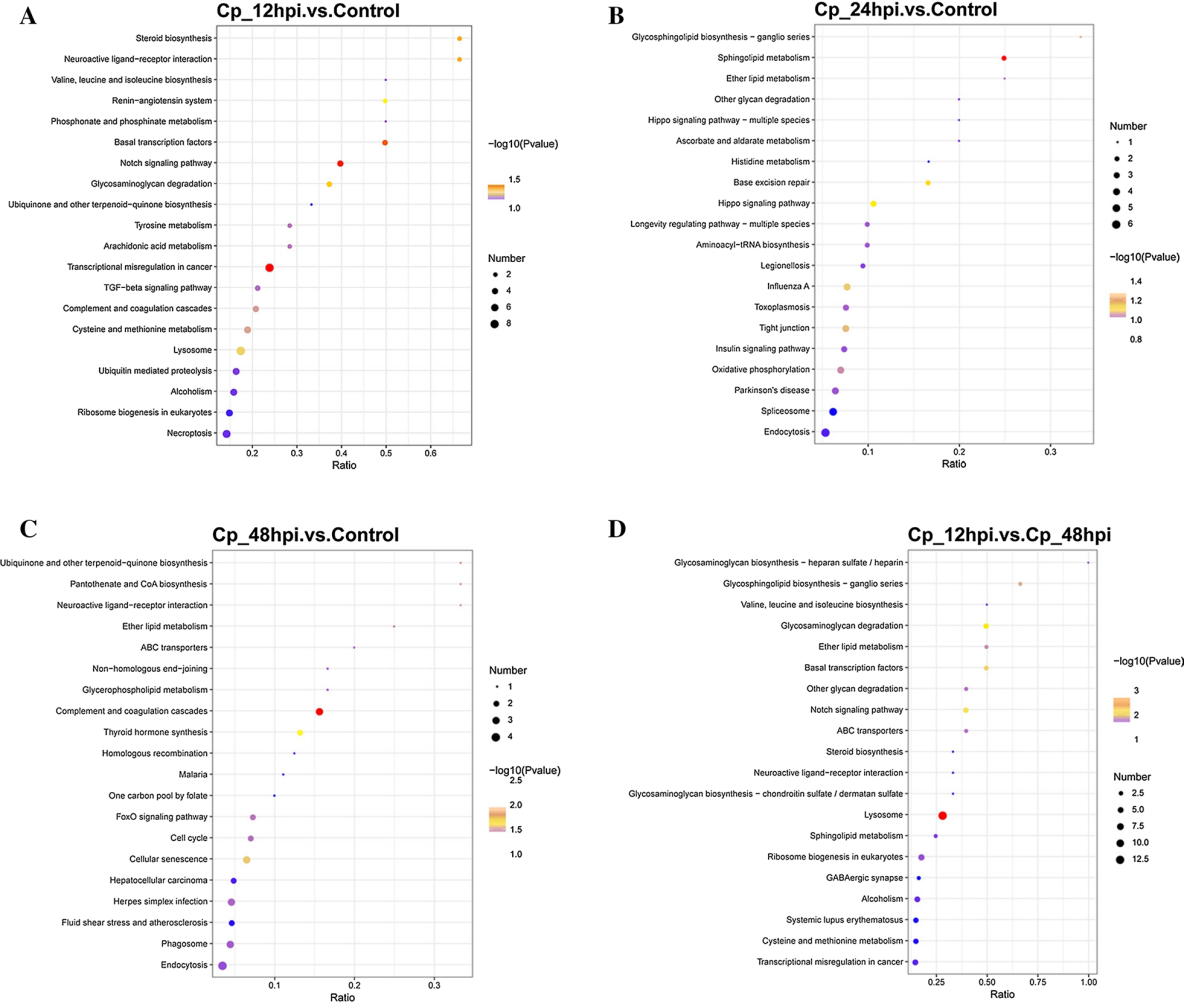
KEGG pathway analysis of differentially expressed proteins. **a**–**d** KEGG pathway analysis was performed on the differentially expressed proteins in Cp_12hpi vs. control (**a**), Cp_24hpi vs. control (**b**), Cp_48hpi vs. control (**c**) and Cp_12hpi vs. Cp_48hpi (**d**). The abscissa in the figure is the ratio of the number of different proteins in the corresponding pathway to the total number of proteins identified by the pathway. The color of the dot represents the Pvalue value of hypergeometric test. The color ranges from blue to red. The redder the color is, the smaller the value is. The size of the point represents the number of differential proteins in the corresponding pathway

For achieving different specific biological functions, gene products need to coordinate with each other in an orderly manner. That is why the abundant pathway information in KEGG database will help us to know the functions of proteins at the system level, such as metabolic pathways, genetic information transmission and some complex biological functions such as cell processes. The top 20 pathways are displayed in Fig. 4. For example, of the differentially expressed proteins, Steroid biosynthesis, Neuroactive ligand-receptor interaction, Valine, leucine and isoleucine biosynthesis, Renin-angiotensin system and the others were in the BVDV NADL-infected MDBK cells at 12 hpi (Fig. 4a). Glycosphingolipid biosynthesis-ganglio series, Sphingolipid metabolism, Ether lipid metabolism, Other glycan degradation and the others were involved in the BVDB NADL-infected MDBK cells at 24 hpi (Fig. 4b). Ubiquinone and other terpenoid-quinone biosynthesis, Pantothenate and CoA Biosynthesis, Neuroactive ligand-receptor interaction, Ether lipid metabolism and the others were involved in the BVDV NADL-infected MDBK cells at 48 hpi (Fig. 4c). Compared with the MDBK cells infected for 48 h, Ascorbate and aldarate metabolism, Steriod biosynthesis, and Histidine metabolism were contained in the BVDB NADL-infected MDBK cells at 24 hpi (Fig. 4d).

### Subcellular localization analysis of differentially expressed proteins

**Fig. 5:**
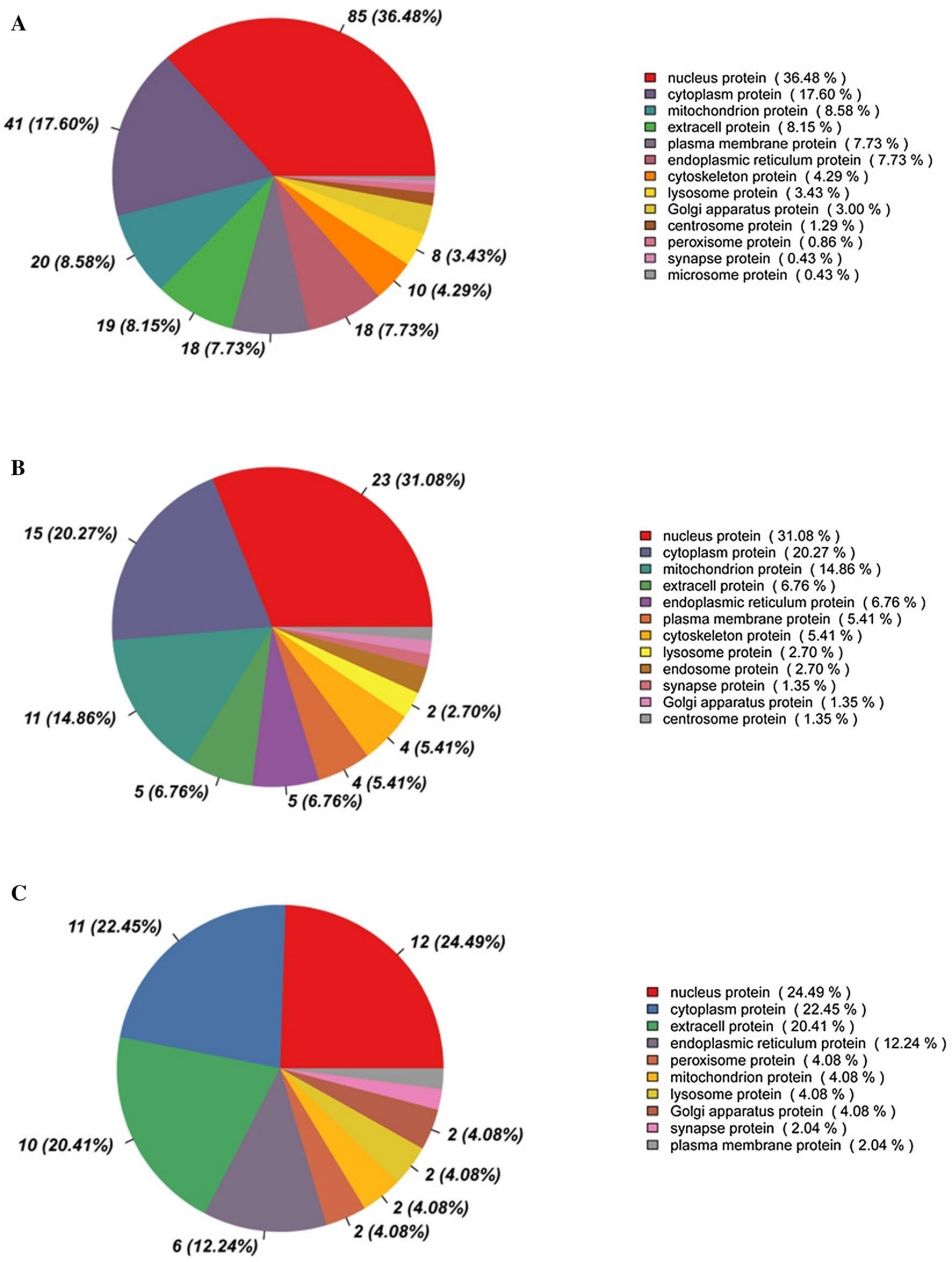
Subcellular localization analysis of differentially expressed proteins. **a**–**c** Analysis of the subcellular localization of the differently expressed proteins at 12 hpi (**a**), 24 hpi (**b**), and 48 hpi (**c**)

Among the proteins that show significantly different levels of expression after infection, 357, 101, and 66 proteins were differentially expressed. The statistical analysis of the subcellular localization proportion of the different proteins of each comparison pair was carried out, as shown in Fig. 5. The results showed at 12 hpi after BVDV NADL infection, 36.48% of the differentially expressed proteins were nuclear localization, 17.60% were cytoplasmic localization, 8.85% were mitochondrial localization, 8.15% were extracellular localization, and 7.73% were endoplasmic reticulum localization (Fig. 5a). For the differentially expressed proteins at 24 hpi, there were 31.08% in the nucleus, 20.27% in the cytoplasm, 14.86% in the mitochondrion and 6.67% in the extracell(Fig. 5b). For the differentially expressed proteins at 48 hpi, there were 24.49% in the nucleus, 22.45% in the cytoplasm, 20.41% in the extracell and 12.24% in the endoplasmic reticulum (Fig. 5c).

### Protein–protein interaction network

**Fig. 6:**
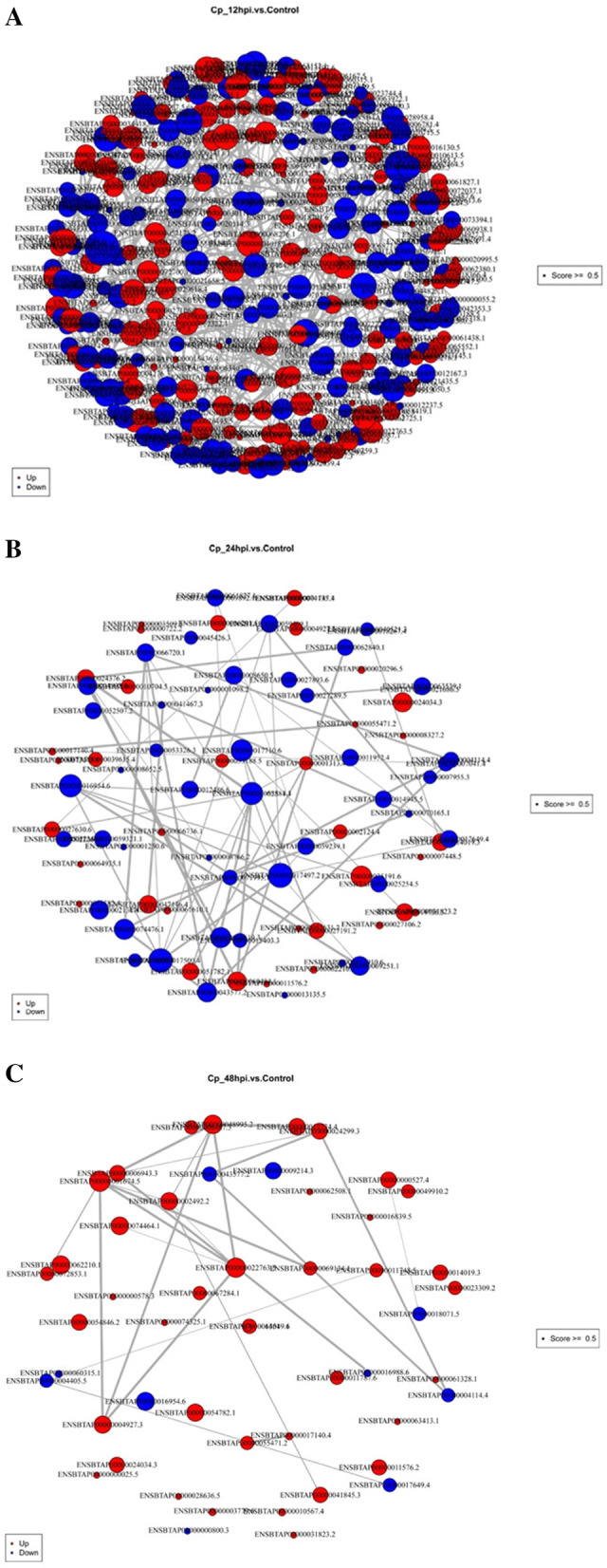
Protein–protein interaction network. **a**–**c** Protein–protein interaction network of the differently expressed proteins at 12 hpi (**a**), 24 hpi (**b**), and 48 hpi (**c**). Each node in the interaction network represents a protein, and the size of the node represents the number of proteins interacting with it. The larger the node, the more proteins interacting with it. The red nodes indicate up-regulated proteins and blue nodes indicate down-regulated proteins

We used the STRING database to perform network analysis on differentially expressed proteins at different periods after BVDV NADL infection to understand their interactions in the pathogenesis of BVDV. The interaction between these different proteins can be visualized by cytoscape software. From the networks, we can intuitively discover that some proteins called key protein molecules interact with many other proteins and know the importance of certain proteins (Fig. 6a–c).

The results showed that the regulatory cytokines were different at different stages after BVDV NADL-infection. FAU ubiquitin like and ribosomal protein S30 fusion (FAU), heat shock protein family A (Hsp70) member 8 (HSPA8), plasminogen (PLG) had the most interaction at 12, 24, and 48 hpi, respectively. At 12 hpi, FAU which is involved in the ribosome pathway interacts with 7 significantly different proteins (Fig. 6a). While HSPA8 as a member of the MAPK signaling pathway cytokines interacts with 8 other down-regulated proteins at 24 hpi. And there is a group of strongly interacted proteins which is involved in RNA processing, nucleic acid binding, negative regulation of transcription from RNA polymerase II promoter and protein binding in response to BVDV NADL infection, including PTBP1-HNRNPM-HSPA1A-PRPF8-HEXIM1-HNRNPUL1 (Fig. 6b). At 48 hpi, PLG involved in the neuroactive ligand-receptor interaction pathway interacts with 11 up-regulated proteins. And two groups of proteins interacted with each other strongly, including PLG-CCNB2-KNG1-ALB-SERPINA1, which are involved in proteolysis and cysteine-type endopeptidase inhibitor activity, and PLG-KNG1-CCNB2-PRNP-HNRNPUL1-SRSF2-PRPF8, which are involved in serine-type endopeptidase activity, proteolysis, protein homooligomerization, and nucleic acid binding (Fig. 6c).

## Discussion

MDBK cells are currently widely used in the study of BVDV, and few researchers have studied the proteomics of BVDV infected MDBK cells. The research and development of BVDV is in a relatively lagging state. By studying the differentially expression proteins of BVDV-infected MDBK cells, we can systematically understand the interaction between the virus and host cells, looking for host cell immune regulation related proteins to BVDV infection. Furthermore, this study can also provide new ideas for the prevention and control of BVDV and a reference for the research of other viruses of the genus Pestivirus. So far, BVDV has lots of various subtypes through research in the world [13]. Because of the difficulty of studying all these types, we choose BVDV-1 NADL, commonly used in the Chinese animal husbandry [14].

Currently, various proteomic methods have been widely used to study the interaction between virus and host cell [8, 15]. Isobaric Tags for Relative and Absolute Quantitation (iTRAQ) is a peptide in vitro labeling technology developed by American Applied Biosystems ABI. As a new proteome quantification technology, iTRAQ can perform more accurate and effective high-throughput proteome analysis than traditional protein qualitative technology. In recent years, iTRAQ-based proteomics technology has been applied to virology research several times.

The interaction between virus and host cell is a very complex process, involving a large number of protein expression changes and signal transduction [16]. Here, iTRAQ coupled with tandem mass spectrometry (LC–MS/MS) was applied to analyze the protein expression profiles of MDBK cells which were infected with BVDV NADL at 3 different time points. In this study, a total of 357, 101, and 66 DEPs were identified at 12, 24, and 48 hpi based on a fold change > 1.5 or < 0.67 and p value < 0.05. From our results, a significant difference was found in proteins in host cells. This suggests that the DEPs play a significant regulatory role in BVDV NADL-infected MDBK cells. Massive proteins were up-regulated during each period. Simultaneously, the up-regulated proteins were displayed much more than the down-regulated proteins, and this appears clearly at 48 hpi. This suggests the possibility for the host at different time points to be against BVDV NADL infection. Further analysis of the function of these proteins can help to understand the relationship between MDBK cells and BVDV NADL. It provides a theoretical basis for the response of host cells to BVDV NADL infection and the development of vaccine.

Functional analysis of these dysregulated proteins based on GO annotation showed that GO terms were completely different at 12 hpi, 24 hpi and 48hpi in order. It also showed that infected cells respond differently at various time points of BVDV NADL infection. Actually, a large number of the differentially expressed proteins are concentrated in the regulation of biological process (BP) and cellular process and signal transduction at 12 hpi. While at 24 hpi, the most enriched GO terms were cell surface receptor signaling pathway, intermediate filament and extracellular space. However, in the later stage of infection, there was no significant enrichment of differentially expressed proteins in the biological process. GO annotation and pathway analysis revealed that these proteins are being in a variety of cellular biological processes, containing metabolic pathways, protein processing in the endoplasmic reticulum, ECM receptor interactions, and actin cytoskeleton rules. By protein interaction network analysis, we speculate that the MAPK signaling pathway may play a role in BVDV NADL-infected cells. These results of this study lay a foundation for further research on the pathogenesis of BVDV and the selection of antiviral targets.

1. Differentially expressed proteins involved in immunity system

Non-specific immunity system refers to the body's innate normal physiological defense function, which can make corresponding immune responses to the infection of various viruses. Pattern recognition receptors (PRRs) are the representative of immune receptors in innate immunity and can recognize viral RNA in the cytoplasm [17]. It actually plays an important role in anti-viral natural immunity [18]. In this study, lots of the DEPs were involved in the Toll-like receptor(TLR), nucleotide binding oligomerization domain (NOD)-like receptor (NLR) and MAPK signaling pathways, including ring finger protein 31 (RNF31), ANTXR cell adhesion molecule 2 (ANTXR2), tumor protein p53 binding protein 1 (TP53BP1), caspase 8 (CASP8), and insulin receptor (INSR). The MAPK family plays a regulatory role in physiological and pathological processes such as cell growth, differentiation and inflammation. And many studies have shown that the RLR (retinoid acid-inducible gene-Ι(RIG-I)-like receptor), TLR and NLR signaling pathways play significant regulatory roles in Flavivirus-infected cells [19, 20].

We found that the expression of some proteins involved in the immune response was altered following BVDV NADL infection such as interferon regulatory factors (IRFs). IRFs are a kind of multifunctional transcription factors, which can specifically bind to interferon (IFN) gene promoter and interferon stimulatory response elements (ISRE) in interferon stimulation response gene (ISG). Therefore, IRFs have important significance in the body's anti-viral infection and regulating cell growth. From our research, the data suggested that the expression of interferon regulatory factor 2 (IRF2) were meaningfully upregulated. IRF2 is reported to be an important regulator of breast cancer invasion and metastasis; however, its role in the antiviral process and immune regulation needs to be explored in further studies.

2. Differentially expressed proteins involved in cytoskeleton and ECM-receptor interactions

Cell morphology, cell movement and intercellular adhesion are maintained by cytoskeletal proteins. In addition, the cytoskeleton also plays a key role in organelle location and vesicle transport. It is worth mentioning that the cytoskeletal network also plays the role of promotion in the process of virus infection. After the host cell is infected by the virus, its cytoskeleton will break or even disintegrate [21]. Of the 8 DEPs in the regulation of actin cytoskeleton, integrin subunit beta 5 (ITGB5), ENAH actin regulator (ENAH) and Rho guanine nucleotide exchange factor 7 (ARHGEF7) are significantly up-regulated.

In the present study, integrin subunit beta 5 (ITGβ5), collagen type IV alpha 1 chain (COL4A1) and syndecan 1 (SDC1) are significantly up-regulated. The integrin acts as a transmembrane joint between the extracellular matrix cell and the intracellular actin skeleton, which connects the extracellular matrix with the intracellular skeleton network into a whole. A series of related studies have shown that the abnormal expression of ITGβ5 is closely related to the pathological process of various diseases. ITGβ5 can promote the formation of blood vessels in various diseases. It can also interact with endothelial growth factor receptor 2 (VEGF2) to inhibit the apoptosis of cells. Collagens are the most abundant and widely distributed functional proteins in mammals and play a significant role in cell movement, angiogenesis, and tissue formation or repair. Collagens are also main components of the ECM which were associated with many other diseases, including Ehlers-Danlos syndrome, Kniest dysplasia, Alport syndrome, and certain arterial aneurysms [22]. Whether the different types of collagens have many different kinds of functions in BVDV NADL infection, that is still not clear. After BVDV NADL-infection, we found a lot of significant pathological changes in the cells with viral titer at 48 hpi. However, the experimental results do not indicate that the proteins related to the cytoskeleton and ECM-receptor interactions in MDBK cells could promote the proliferation and release of BVDV NADL.

3. Differentially expressed proteins involved in endoplasmic reticulum protein processing

Endoplasmic reticulum (ER) is the most important part for protein synthesis and maturation. The endoplasmic reticulum is found in many molecular chaperones and helps proteins fold and assemble [23]. Lots of studies have shown that viral infection not only alters the endoplasmic reticulum, but also activates the unfolded protein response (UPR), thereby promoting viral replication [24, 25]. In our study, 14 DEPs were in endoplasmic reticulum protein processing. Among these proteins, endoplasmic reticulum lectin 1 (ERLEC1) and lectin and mannose binding 1 (LMAN1) are both up-regulated at 48 hpi. Moreover, ubiquitin conjugating enzyme E2 J1 (UBE2J1) is up-regulated at 12 hpi. The results showed that DnaJ heat shock protein family (Hsp40) member A2 (DNAJA2), heat shock protein family A (Hsp70) member 8 (HSPA8) and heat shock protein family A (Hsp70) member 1A (HSPA1A) are down-regulated at 24 hpi, while DnaJ heat shock protein family (Hsp40) member B4 (DNAJB4) is significantly up-regulated at 12hpi. Heat shock protein (HSP) is a kind of highly conserved protein family whose expression is inhibited by the body or cells under normal conditions and stimulated by adverse environmental factors [26]. It has various biological functions including cell protein self-stabilization, anti-apoptosis, anti-oxidation, cooperative immunity, etc. For example, HSP70 is highly expressed in various DNA virus and RNA virus infections and inhibits virus replication [27, 28]. The decreased activity of heat shock proteins will lead to the development of inherited peripheral motor neuropathy, Alzheimer's disease and other diseases [29]. This study identified that HSPA8 and HSPA1A were significantly down-regulated at 24 h after BVDV NADL infection, which may cause the host to display abnormal neurological symptoms. Since heat shock proteins are involved in protein folding, the activation of UPR signals in the endoplasmic reticulum stress response may play a role in specific viral functions. Therefore, endoplasmic reticulum stress proteins may also be important for the replication of many viruses [25]. The entry of the virus into the target cell is one of the most important steps in virus reproduction, so further study is needed to verify the specific role of these differential proteins after BVDV NADL infection.

4. Differentially expressed proteins involved in translation

Most translation-related proteins are up-regulated during BVDV NADL infection. Among the ribosomal proteins, Treacle ribosome biogenesis factor 1(TCOF1), nuclear transport factor 2 like export factor 2(NXT2), ribosomal protein L37(RPL37), ribosomal protein L29 (RPL29) and mitochondrial ribosomal protein S18A(MRPS18A) are all up-regulated in the early stages of infection. As the translation initiation factor, eukaryotic translation initiation factor 4E binding protein 1 (EIF4EBP1), eukaryotic translation initiation factor 4E (EIF4E) and eukaryotic translation initiation factor 3 subunit F (EIF3F) are up-regulated in the course of BVDV NADL-infection. We hypothesized that BVDV NADL would use the MDBK cells' protein synthesis system to produce substantial viral proteins.

5. Differentially expressed proteins involved in metabolic processes

Studies have shown that cell metabolism in host cells can be significantly changed by viral infection [30, 31]. In our research, we found many proteins which were involved in metabolic processes expressed variously in the BVDV NADL-infected MDBK cells. And 59 differentially expressed proteins (9.14%) were sorted as metabolic pathways. These proteins are mainly involved in glycolysis, pyruvate metabolism, and citrate cycle (TCA cycle). Among these proteins in the metabolic pathways, pyruvate dehydrogenase E1 beta subunit (PDHB), aldehyde dehydrogenase 7 family member A1 (ALDH7A1), aldehyde dehydrogenase 3 family member A2 (ALDH3A2) and pyruvate kinase M1/2 (PKM) are involved in both the glycolysis and pyruvate metabolism. Aldehyde dehydrogenase 7 family member A1 (ALDH7A1) and pyruvate kinase M1/2 (PKM) are down-regulated at 24 hpi. Pyruvate dehydrogenase E1 beta subunit (PDHB), citrate synthase (CS), ATP citrate lyase (ACLY) and isocitrate dehydrogenase (NAD( +)) 3 non-catalytic subunit gamma (IDH3G) are involved in the citrate cycle (TCA cycle). At present, the interaction mechanism of virus-host metabolism needs to be further studied. In particular, how the key genes of the metabolic pathway regulate viral susceptibility is worth considering. This process provides a new direction for this study. Antiviral drugs can be studied by studying how protein expression in metabolic pathways inhibits virus generation.

## Conclusion

In summary, the differentially expressed proteins were identified in BVDV NADL-infected MDBK cells through iTRAQ analysis. More importantly, we described and discussed BVDV NADL-infection-associated pathways and proteins which were based on bioinformatics analysis. Although the roles of the proteins that were identified in this study were not studied, it suggested that all or part of them are being in host-virus interactions. Consequently, this analysis about the responses of the MDBK cells to BVDV infection provides valuable information for a better understanding of the pathogenesis of BVDV as well as other flaviridae viruses.

## Supplementary Information

**Additional file 1**: **Table S1**. The results of different proteins in the BVDV NADL-infected MDBK cells at 12, 24, and 48 h after infection using iTRAQ technology.

**Additional file 2**: **Table S2**. The results of significantly differentially expressed proteins (p < 0.05 and FC > 1.5 or < 0.67 ([fold change, FC]) in the BVDV NADL-infected MDBK cells at 12, 24, and 48 h after infection using iTRAQ technology.

## Data Availability

The datasets generated during and/or analysed during the current study are available from the corresponding author on reasonable request.

## Abbreviations

BVD: Bovine viral diarrhea
BVDV: Bovine viral diarrhea virus
DMEM: Dulbecco’s modified Eagle’s Medium
MDBK: Madin-Darby bovine kidney
PBS: Phosphate buffered saline
FBS: Fetal bovine serum
TCID_50_: Tissue culture infective dose
BP: Biological process
CC: Cellular component
GO: Gene ontology
iTRAQ: Isobaric tags for relative and absolute quantitation
KEGG: Kyoto encyclopedia of genes and genomes
LC–MS/MS: Liquid chromatography-tandem mass spectrometric
